# Strengthening malaria microscopy using artificial intelligence-based approaches in India

**DOI:** 10.1016/j.lansea.2022.100054

**Published:** 2022-08-03

**Authors:** Shrikant Nema, Manju Rahi, Amit Sharma, Praveen Kumar Bharti

**Affiliations:** a ICMR-National Institute of Malaria Research, Sector 8, Dwarka, 110077 New Delhi, India; b Division of Epidemiology and Communicable Diseases, Indian Council of Medical Research, New Delhi, India

Malaria is primarily diagnosed by rapid diagnostic test kits (RDT) and microscopy. Microscopy has become a field standard (around 11 million blood smear examinations carried out in India in 2021)^1^ and the most frequently used diagnostic test, since the inception of a structured malaria control program in India.^2^ RDT is another versatile tool used for diagnosis in malaria-endemic areas where microscopy is not feasible. However, the currently used histidine-rich protein-2 (HRP-2) as a diagnostic target has shown deletion in the *Plasmodium falciparum* parasite^3^ and longer persistence of HRP-2 in the blood after successful treatment affects the RDT performance.

Microscopy has also reported variable sensitivity and specificity in different clinical and transmission settings. Nevertheless, microscopy has become a fundamental tool to support clinical research, malaria case management, and monitoring of anti-malarial drug efficacy. However, the outcomes of microscopic diagnosis are time-consuming and depend on the quality of blood smear, staining, and fixation. A skilled microscopist, a good quality microscope, a stable supply chain, and access to power sources in remote places are major limitations, which necessitate the need for training, expertise, and well-organized workflows. There is heterogeneity even among trained technicians, which makes valid comparisons over time and space difficult,^4^ and its accuracy is hampered in the case of low parasitemia and mixed-species infection.^5^ One-time malaria parasite testing underestimates microscopic diagnosis by 12%, which can be improved by repeated sampling for up to 12 h to confirm the results.^6^ Studies also show that microscopy misses more than 25% of malaria cases.^5^ Such limitations are exaggerated in the remote, inaccessible, and hard-to-reach regions of India, which are highly malarious.

The above limitations entail the necessity of developing AI-based microscopic approaches by formulating and using locally annotated databases of malaria parasite images. Deep learning models such as fully connected neural networks, convolutional neural networks, convolutional encoder-decoder, U-Nets, and generative adversarial networks are primarily focused on three key areas: image segmentation, image enhancement, and particle tracking,^7^ All these have shown extraordinary performance and are widely applied in microscopic image analysis due to their high speed, accuracy, flexibility, and low cost. The features of malaria parasite images can be extracted from a quality-assured biomedical image repository in the form of a set of reference slides. Texture and intensity-based information can be retrieved from infected and non-infected erythrocytes using the Haralick texture feature and the Grey level run length to characterize the malaria parasite-infected blood smear image. Furthermore, microscopists may not be adept at recognising distinct stages of malaria parasites in blood smears (*e.g.,* gametocytes) due to a lack of training and expertise. Though this may not be needed at peripheral levels for decision-making in treatment, but an understanding of species-specific stages and morphology is important to prevent misdiagnosis. Deep learning-based malaria microscopy can overcome this barrier. Additionally, smartphones may also play an important role in the development of parasite detection algorithms, decreasing the need for highly skilled microscopists.^7^ Furthermore, malaria microscopy quality assurance in the form of regular cross-checking of examined blood smears has several operational and technological issues. A collection of well-depicted, good-quality blood films is a vital asset to a malaria quality assurance system and is also needed for training the algorithm systems in the AI-based approach. Though the World Health Organization (WHO) has recommended a regional malaria slide bank (national reference slide sets)^2^ but it is difficult for a majority of countries to develop their slide bank due to a lack of funding and limited access to positive blood samples.^8^

Here, integrating AI-based approaches to develop a malaria slide bank would be helpful even in the post-elimination period. The AI-powered microscope has already proven that it can detect malaria parasites effectively enough to meet WHO microscopy standards.^9^ The AI-based microscopes could be particularly useful in detecting drug-resistant parasite strains spreading throughout Southeast Asia by detecting and clustering drug-induced morphological outliers by the mechanism of action.^10^ Anti-malarial drug resistance monitoring uses highly reliable microscopy to determine how quickly malaria drugs have reduced the number of parasites in the blood. Machine learning could improve endemic area accuracy and consistency, allowing countries to implement monitoring more effectively. Furthermore, the clinical impact of low parasite density infections continues to challenge diagnostic accuracy. A deep learning-based analysis after staining with 4′,6-diamidino-2-phenylindole fluorogen could be useful in detecting low-level parasitemia.^11^ The scientific community has acknowledged the emergence of AI-based approaches and integrating them with the malaria elimination strategy would gear up the elimination efforts.

Year 2022, the world has marked Malaria Day under the theme “Harnessing innovation to reduce the malaria disease burden”. Integrating innovations such as AI-based approaches into the current microscopy method would strengthen surveillance via augmenting the capacities of healthcare facilities, which is required to increase the use of quality-assured microscopy. However, the implementation of AI-based microscopy requires continuous surveillance and supervision to avoid gaps in AI-building blocks, infrastructure in rural India, and disruption in physician–patient relationships. Over the next decade, investments in artificial intelligence-based microscopy approaches will help to improve diagnostic sensitivity and accuracy, which is the prerequisite for malaria elimination.

## Contributors

SN, PKB: conceptualization, methodology. SN, MR, AS, PKB: writing the original draft, reviewing and editing.

## Declaration of interests

Authors declare no competing interests.
